# Walk Score, Transportation Mode Choice, and Walking Among French Adults: A GPS, Accelerometer, and Mobility Survey Study

**DOI:** 10.3390/ijerph13060611

**Published:** 2016-06-20

**Authors:** Dustin T. Duncan, Julie Méline, Yan Kestens, Kristen Day, Brian Elbel, Leonardo Trasande, Basile Chaix

**Affiliations:** 1 Department of Population Health, New York University School of Medicine, New York, NY 10016, USA; dustin.duncan@nyumc.org (D.T.D.); brian.elbel@nyumc.org (B.E.); Leonardo.Trasande@nyumc.org (L.T.); 2 College of Global Public Health, New York University, New York, NY 10016, USA; 3 Population Center, New York University, New York, NY 10016, USA; 4 Center for Data Science, New York University, New York, NY 10016, USA; 5 Inserm, UMR-S 1136, Pierre Louis Institute of Epidemiology and Public Health, Nemesis Team, Paris 75012, France; julie.meline@inserm.fr; 6 Sorbonne Universités, UPMC Unv Paris 06, UMR-S 1136, Pierre Louis Institute of Epidemiology and Public Health, Nemesis Team, Paris 75012, France; 7 Department of Social and Preventive Medicine, École de Santé Publique de l’Université de Montréal, Montreal, Montréal, QC H3N 1X9, Canada; yan.kestens@umontreal.ca; 8 Department of Technology, Culture and Society, New York University Tandon School of Engineering, New York, NY 11201, USA; kday@nyu.edu; 9 Wagner School of Public Service, New York University, New York, NY 10012, USA; 10 Departments of Pediatrics and Environmental Medicine, New York University School of Medicine, New York, NY 10016, USA

**Keywords:** built environment, walkability, walking trips, Walk Score, physical activity, transportation, GPS, accelerometer, Paris

## Abstract

Background: Few studies have used GPS data to analyze the relationship between Walk Score, transportation choice and walking. Additionally, the influence of Walk Score is understudied using trips rather than individuals as statistical units. The purpose of this study is to examine associations at the trip level between Walk Score, transportation mode choice, and walking among Paris adults who were tracked with GPS receivers and accelerometers in the RECORD GPS Study. Methods: In the RECORD GPS Study, 227 participants were tracked during seven days with GPS receivers and accelerometers. Participants were also surveyed with a GPS-based web mapping application on their activities and transportation modes for all trips (6969 trips). Walk Score, which calculates neighborhood walkability, was assessed for each origin and destination of every trip. Multilevel logistic and linear regression analyses were conducted to estimate associations between Walk Score and walking in the trip or accelerometry-assessed number of steps for each trip, after adjustment for individual/neighborhood characteristics. Results: The mean overall Walk Scores for trip origins were 87.1 (SD = 14.4) and for trip destinations 87.1 (SD = 14.5). In adjusted trip-level associations between Walk Score and walking only in the trip, we found that a walkable neighborhood in the trip origin and trip destination was associated with increased odds of walking in the trip assessed in the survey. The odds of only walking in the trip were 3.48 (95% CI: 2.73 to 4.44) times higher when the Walk Score for the trip origin was “Walker’s Paradise” compared to less walkable neighborhoods (Very/Car-Dependent or Somewhat Walkable), with an identical independent effect of trip destination Walk Score on walking. The number of steps per 10 min (as assessed with accelerometry) was cumulatively higher for trips both originating and ending in walkable neighborhoods (*i.e.*, “Very Walkable”). Conclusions: Walkable neighborhoods were associated with increases in walking among adults in Paris, as documented at the trip level. Creating walkable neighborhoods (through neighborhood design increased commercial activity) may increase walking trips and, therefore, could be a relevant health promotion strategy to increase physical activity.

## 1. Introduction

Substantial research has shown that physical activity is associated with numerous health benefits, such as obesity prevention, reducing risk for cardiovascular disease, type 2 diabetes and certain cancers, and improvements in mental health and sleep [1,2,3]. According to the World Health Organization and European Union Working Group on Sport and Health, the public health goal is 30 min of physical activity on most days [4,5]. Many people, however, do not meet the public health physical activity recommendations. For example, in France, one study found that 62% of men and 52% of women only met recommended levels of physical activity [6].

Studies have shown that walkable built environment features (e.g., sidewalks, recreational open spaces) are associated with increased levels of physical activity [7]. While individual built environment features have been associated with physical activity outcomes, some recent research has found that overall neighborhood walkability (measured by composite measures of neighborhood walkability, such as Walk Score–yielding a 0 to 100 normalized scale), is the most predictive of physical activity outcomes [8]. Walk Score has been associated with physical activity outcomes, especially walking across geographic contexts [9,10,11,12,13,14,15,16,17,18,19,20]. For example, using data from the six-city United States (U.S.) Multi-Ethnic Study of Atherosclerosis (MESA), one study found that a higher Walk Score was associated with lower odds of not walking for transport and with more minutes/week of transport walking. Compared to those in a “Walker’s Paradise”, lower categories of Walk Score were associated with a linear increase in odds of not transport walking and a decline in minutes of leisure walking [9]. In a sample of recent Cuban immigrants who lived in neighborhoods across Miami-Dade County, FL (U.S.), another study found that for each 10-point increase in Walk Score, there was a significant 19% increase in the likelihood of purposive walking, a 26% increase in the likelihood of meeting physical activity recommendations by walking, and 27% more minutes walked in the previous week [10]. In addition, one recent study found that Walk Score was associated with walking for transport, but not recreational walking nor total walking among a nationally-representative sample of American adults [11]. Among a large representative sample of Australian adults aged 18–64 years, a recent study found that residents in highly and somewhat walkable areas were twice and 1.4 times more likely to accumulate 30 min of walking per day compared to those in very car-dependent neighborhoods, respectively [12]. In addition, the mean duration of walking was also longer for participants living in highly and somewhat walkable areas compared to those in very car-dependent areas. Finally, a recent study among a representative sample of Canadian adults found that increased Walk Score was associated with increased odds of utilitarian walking, but no associations were found with daily steps assessed via accelerometer [13].

While Walk Score is a valid measure of estimating certain aspects of neighborhood walkability and is an up-to-date walkability tool [8], limited Walk Score research has been conducted in certain geographies. There is a need for research that examines the utility of Walk Score in the European context, for example. The vast majority of the existing Walk Score research has been conducted in the United States, although studies are now beginning to be conducted in other geographic locations, such as Canada, as previously mentioned. Furthermore, many Walk Score studies rely on self-reported potentially recall-biased physical activity outcomes [3]. Non-differential misclassification is physical activity outcomes due to self-report may explain the absence of associations found in some studies on Walk Score and physical activity outcomes [14,15,16]. Use of objectively measured physical activity data is therefore critical. In addition, the majority of studies of the association between walkable environments and physical activity (including Walk Score studies) have examined only the residential environment, thus missing other places where walking may occur. Use of new geospatial technology (e.g., global positioning system (GPS) technology) as opposed to defining a neighborhood using a residential administrative area (e.g., census tract) minimizes spatial misclassification [21] and addresses the uncertain geographic context problem [22,23,24]. More specifically, GPS technology can allow us to precisely understand daily mobility, the location of where physical activity occurs, and which daily life environments are more particularly associated with walking [25]. While some studies have used GPS technology in examining associations between neighborhood walkability and physical activity [26,27,28,29], the usefulness of Walk Score still needs to be confirmed using GPS tracking technology through more fine-grain analyses. Such fine-grain analyses relying not only on GPS technology but also GPS mobility surveys could evaluate association at the trip level considering trip-level environmental factors, which is important to contextualize walking in its immediate environments [30]. In addition, few studies have examined the influence of neighborhood walkability on transportation mode choice, which can relate to physical activity and overall health, including cardiometabolic health. 

As such, the purpose of the current study is to examine associations between composite neighborhood walkability (measured by Walk Score), transportation mode choice, and walking among adults in Paris, France. In this study, we conducted a trip-level analysis, focusing on the trip origin and the trip destination, which is the focus of the next generation of spatial mobility research [30]. In the French RECORD (Residential Environment and CORonary heart Disease) Study, walking was assessed both with accelerometers and by combining GPS tracking with a GPS-based mobility survey, allowing for a more comprehensive assessment of Walk Score influences on walking and transportation mode choice. 

## 2. Methods

### 2.1. Study Population and Geospatial Data

#### 2.1.1. Study Population

The RECORD participants were recruited during preventive health checkups in 2007–2008 and 2011–2014. They were born in 1928–1978 and were residing (at baseline) in 112 municipalities of the Paris Ile-de-France region, so participants come from throughout the Paris metropolitan area [31]. In the second study wave, after completing computerized questionnaires at the IPC Medical Centre [32], 410 participants were invited to enter in 2012–2013 in the RECORD GPS Study (approved by the French Data Protection Authority (ethical approval: DR-2011-421, 21 October 2011) [33]. Of these, 247 accepted to participate and signed an informed consent form. Nine participants withdrew from the study, the data collection failed for two participants, and data were incomplete for two participants, resulting in a final participation and completion rate of 57.1% (*n* = 234). Seven participants were then removed because they lived out of the Ile-de-France region, or spent their 7-day follow-up far from their residence. The analytic sample included 7440 trips made by 227 individuals over 7 days.

In a previous study [30], we compared the participants who accepted to take part in the GPS study and completed the protocol to those who refused to participate, using a binomial regression model estimated among the 410 participants who were invited to enter the study with accepting to participate in the study or not as the outcome. Among participants who were proposed to enter the GPS study, age and gender were not associated with participating in the study. There was a trend of association indicating that the unemployed participants had a lower probability to participate in the study. No other characteristic was associated with the probability of participation.

#### 2.1.2. Collection and Processing of GPS and Accelerometer Data

Participants wore a QStarz BT-Q1000XT GPS receiver (Taipei, Taiwan) and an Actigraph GT3X+ tri-axial accelerometer (The Actigraph, Pensacola, FL, USA) on the right hip with a dedicated belt for 8 days (the recruitment day and 7 additional days), which we have used in our past research [33]. The recruitment day was not included in the period of the study. The 7 days start the day after the recruitment day. Supporting information for the electronic mobility survey was collected by instructing the participants to complete a travel diary of their activity locations (with arrival and departure times) over the data collection period (the travel diary was used to support the subsequent telephone mobility survey but was not the survey itself). 

The GPS data (one point every 5 seconds) were processed prior to the mobility survey by an ArcInfo 10 Python script (ESRI, Redlands, CA, USA) [34]. The goal was to identify the participants’ activity locations (any type of activity at a stationary location) over the data collection period. The algorithm calculated a kernel density surface based on the set of GPS points for each participant, extracted peaks as potentially visited locations, and derived a list of all visits over the period made to each detected location with their start and end times [34]. Thus the start and end times of each trip were identified.

ActiLife 5.10 (The Actigraph, Pensacola, FL, USA) (with default settings) was used to identify episodes of nonwear of the accelerometer (floating windows of consecutive 5 s epochs with a 3-axes count equal to 0 for at least 60 min with a Spike tolerance of 2 min of nonzero epochs). Trips that overlapped with nonwear periods were identified. The number of steps estimated by ActiLife was computed for each trip using start and end times.

#### 2.1.3. GPS-Based Mobility Survey

The telephone mobility survey was based on the Mobility Web Mapping application [33]. With the help of the participants and considering the travel diary filled during the follow-up, the survey operator was instructed to geolocate visits to activity locations undetected by the algorithm or for which GPS data were missing (e.g., if the GPS receiver was left at home). The operator could also modify/remove detected visits to locations that were inaccurate. The dates/hours of arrival/departure to/from locations were provided by the algorithm but could also be edited. The information collected for each visit at a given location included: (1) the type of activity conducted and (2) the different transportation modes that were used to arrive at the location in a chronological order. Regarding subway trips: (1) most were underground, but some portions of the trips were over the ground; (2) even in underground trips, it is possible to identify the trip with its start point and end point, even if the itinerary is missed; and (3) in case the trip was entirely missed, in any case, it was retrieved by the survey technician and coded in the database through the web mapping application. 

A detailed timetable over 7 days then was generated by a custom SAS program. This timetable reported: (1) the succession of activity places and trips between places with the start/end times of each episode and (2) the corresponding information on activities and transport modes. 

### 2.2. Transportation Mode Choice and Walking

#### 2.2.1. Walking

Based on the mobility survey, a binary outcome at the trip level was set to 1 (*vs*. 0) if only walking was used for the trip. The number of steps for each 10 min of trip (outcome standardized on the duration of the trip) was expressed as a continuous variable [30]. 

#### 2.2.2. Other Transportation Modes

Three additional binary variables were also defined for the other transportation modes. They were coded to 1 (*vs*. 0) if a bike, public transportation, or a personal motorized vehicle was used in the trip (possibly in addition to other modes). 

### 2.3. Individual/Neighborhood Variables

Age was coded in three categories (35–49; 50–64; 65 years and over). Marital status was coded as living alone or in with a partner. Education was divided into 4 categories: no formal education, primary education, or lower secondary education; higher secondary education and lower tertiary education (1 or 2 years); intermediate tertiary education (3 or 4 years); and upper tertiary education (5 years or more). Household income per consumption unit was coded in three classes based on the tertiles in the sample. Employment status was coded in four categories: stable job; unstable and precarious job; unemployed; and other (including retired individuals). A score of ownership (as a measure of wealth) was based on the following questionnaire item: “Today, are you, you or your household, owner of X (a specific good)?”. The score of ownership was defined by summing the answers (yes coded to 1 *vs*. no) provided for each type of property: (1) secondary residence(s); (2) housing(s) for rent; (3) a shop or a company; and (4) savings or financial investments (>25,000 Euros). The score of ownership was then divided into 3 classes: no property, owner of one type of property, owner of ≥2 types of property. 

Neighborhood socioeconomic status was defined as the proportion of residents aged >20 years with an upper tertiary education (2010 Census) in a circular buffers of 1 mile of radius centered on the residence of the participants. This variable was divided into quartiles comprising a similar number of participants in each. We note that education is one of the strongest determinants for employment and income at the individual and neighborhood levels. Neighborhood education was used because it is a highly relevant covariate in the context of the Paris metropolitan area. All our previous work with the RECORD Study has shown neighborhood education to be a stronger predictor of cardiovascular outcomes than neighborhood income [35,36,37]. Our previous work on built environment effects on walking has also used neighborhood education [38]. We do not control for both neighborhood education and neighborhood income due to multicollinearity. 

### 2.4. Neighborhood Walkability: Walk Score

Walk Score^®^ was originally developed by Front Seat Management, LLC. It calculates neighborhood walkability using a web-based algorithm for a 1-mile radius area around an address. Walk Score uses publicly available data and places added by the Walk Score user community. Walk Score divides facilities into various categories including: educational (e.g., schools), retail (e.g., bookstores), food (e.g., restaurants), recreational (e.g., gyms), and entertainment (e.g., movie theaters). The algorithm uses a distance-decay function. If the closest establishment of a certain type is within 0.25 miles, Walk Score assigns the maximum number of points for that type. No points are awarded for destinations more than 1 mile away. Each destination type is weighted equally. Walk Scores assigned to the various categories are summed and normalized into a continuous score ranging from 0 to 100 (higher scores indicating better walkability). Walk Score has been validated against several features of the built environment (e.g., retail destinations, service destinations, parks, street connectivity, residential density) obtained from Geographical Information Systems (GIS) [39,40,41,42]. Walk Score has also been associated with people’s perception of their built environment (e.g., perceived physical activity facilities) [39]. For this study, Walk Scores were obtained from Walk Score in February 2014 for the geographic coordinates of origin and destination of each trip. Walk Scores were examined continuously and using five categories designated by Walk Score as “Very Car-Dependent” (score below 25, almost all errands require a car); “Car-Dependent” (25–49, a few amenities within walking distance); “Somewhat Walkable” (50–69, some amenities within walking distance); “Very Walkable” (70–89, most errands can be accomplished on foot); and “Walker’s Paradise” (90–100, daily errands do not require a car). 

### 2.5. Statistical Analysis

#### 2.5.1. Analytic Sample

We excluded all the episodes of time spent at activity locations, yielding a sample of 7440 trips. From this main sample, we defined two sub-samples, one for analyzing the walking binary outcome (derived from the survey), and one for analyzing the accelerometry-derived number of steps. For the binary outcome, the following trips were excluded: Atypical trips (e.g., professional tours, *etc*.) (*n* = 8) and trips starting and/or ending outside the Ile-de-France region (*n* = 463). Examples of professional tours include a nurse going from one patient to the other with her car; a truck driver delivering products in several successive supermarkets, *etc.* In transport surveys, usually this is not counted as many different successive trips but as only one trip. The sample for the analysis of the walking outcome (from the survey) comprised 6969 trips. 

For the modeling of the accelerometry outcome (number of steps), the following trips were further removed: trips that overlapped a period of nonwear of the accelerometer (*n* = 397); trips of less than 5 meters of length (*n* = 38); trips of less than 1 min that had a 0 min length in the final database (*n* = 196); and trips with missing accelerometry data (*n* = 25). The analysis of the accelerometry outcome was based on a sample of 6313 trips.

#### 2.5.2. Regression Analyses

First, we conducted descriptive analyses, including frequency, means, and standard deviations for study variables. Then, multilevel logistic and linear models were estimated at the trip level (one observation per trip) [33], with a random effect at the individual level as trips (level 1) were nested within individuals (level 2). These models estimated associations between Walk Score for the trip origin and for the trip destination and the odds of walking in a trip or with the number of steps taken per 10 min of trip. The models were estimated with respectively 6969 and 6313 trips for 227 participants of the Paris region. 

For each sample, the models were adjusted for individual socio-demographic variables and residential neighborhood education. All individual socio-demographic variables were forced into the models. Residential neighborhood education was retained only if it was associated with the outcome. From the multilevel logistic regression models, we report odds ratios for the probability of walking in a trip associated: (1) with a one-point increase in the continuous variable of Walk Score; and (2) with the “Very Walkable” and “Walker’s Paradise” categories in comparison with “Very/Car-dependent/Somewhat Walkable” (categorical variable of Walk Score). For the multilevel linear models, we report beta coefficients expressing increases/decreases in the number of steps taken per 10 min of trip associated: (1) with a one-point increase in the continuous variable of Walk Score; and (2) with the “Very Walkable” and “Walker’s Paradise” categories in comparison with “Very/Car-dependent/Somewhat Walkable”. Consistent with previous Walk Score research [11,12], we combined certain Walk Score categories due to sparse data in them. In particular, the categories “Very Car-Dependent”, “Car-Dependent”, and “Somewhat walkable” were grouped into one category: “Very/Car-Dependent/Somewhat Walkable”. Statistical analyses were conducted with SAS version 9.3 (SAS Institute Inc., Cary, NC, USA).

## 3. Results

The average number of trips (of all models) per person in our sample was 20. Table 1 shows the overall number of trips and the number of trips starting and/or ending at the residence over 7 days (in both cases culminating all motorized and non-motorized modes) according to categories of residential Walk Score. There was a trend indicating that the number of trips per participant increased from less to more walkable residential neighborhoods, although the *p* value was >0.05.

The mean overall Walk Score for all trip origins was 87.1 (SD = 14.5). The mean overall Walk Score for the trip destinations was 87.1 (SD = 14.5). The Walk Score range (maximum minus minimum) for both trip origins and trip destinations was 100.

Table 2 shows the distribution of transport mode choice according to the Walk Score for the trip origin and trip destination. For both the trip origin Walk Score and the trip destination Walk Score, walkable neighborhoods were associated with increased walking and public transportation use, while less walkable neighborhoods were associated with an increased reliance on a personal motorized vehicle (all assessed through the survey). The number of steps per 10 min of trip increased from 221 steps in the least walkable category to 415 steps in the most walkable category of trip origin Walk Scores (*p* < 0.0001).

Table 3 shows adjusted trip-level associations between categorical Walk Score and walking in the trip. A walkable neighborhood at the trip origin and a walkable neighborhood at the trip destination were both independently associated with increased odds of walking in the trip (from the survey). The odds of walking in the trip were 3.48 (95% CI: 2.72 to 4.44) times higher when the trip origin was a “Walker’s Paradise” compared to the least walkable neighborhoods (Very/Car-Dependent-Somewhat Walkable). After mutual adjustment of trip origin and trip destination Walk Scores, the association with the survey walking outcome was of similar magnitude for the trip destination Walk Score categories (OR = 3.48, 95% CI: 2.73 to 4.44). The number of steps taken per 10 min of trip also increased monotonically along walkability categories, independently for the trip origin Walk Score and for the trip destination Walk Score.

Regarding continuous Walk Score, the associations between, on the one hand, Walk Score at the trip origin and Walk Score at the trip destination and, on the other hand, walking in the trip (survey outcome) were positive. The odds of walking were 1.33 (95% CI: 1.26 to 1.41) higher for a 10-point increase in trip origin Walk Score and 1.33 (95% CI: 1.26 to 1.41) higher for a 10 point increase in trip destination Walk Score (mutually adjusted associations, not shown in a Table).

Similarly, independent associations were documented between the continuous Walk Score at the origin and continuous Walk Score at the destination of the trip and the number of steps taken per 10 min of trip. These associations were, respectively, of +19 steps (95% CI: +11 to +26) for a unit increase in the trip origin Walk Score and of +16 (95% CI: +9 to +24) for a unit increase in the trip destination Walk Score (not shown in a Table).

## 4. Discussion

Walk Score is a novel and convenient tool to evaluate neighborhood walkability. In this GPS-based study among a sample of adults in Paris metropolitan area neighborhoods, we found that a walkable neighborhood both at the trip origin and trip destination was associated with increased odds of walking in the trip (assessed in the survey) and with an increased number of accelerometer-assessed number of steps per 10 min of trip. The present study is the largest study to examine relationships between Walk Score and accelerometer-determined walking that used GPS tracking, one of the very few studies that investigated associations between Walk Score and walking at the trip level rather than at the individual level (an important contribution allowing us momentary exposures in each trip rather than overall exposures), and one of few Walk Score studies to be conducted outside of the U.S. 

Several studies have examined associations between Walk Score and physical activity outcomes, especially walking. Our results are consistent with these existing studies. For example, our findings are in line with the other Walk Score study that utilized GPS data [43]. In that study with GPS data collected from 28 older adults with mobility disabilities in King County, Washington (U.S.), participants who took active trips had higher neighborhood Walk Scores than those who did not take active trips [43]. Another study, which used the 2003 Montreal Origin-Destination survey, found that Walk Score was associated with walking trips [44]. Additionally, our findings are similar to a recent study among a representative sample of Canadian adults where increased Walk Score was associated with increased odds of utilitarian walking, except that in this study no associations were found with daily steps assessed via accelerometer [13], contrary to the present work.

In addition to being comparable to existing Walk Score—physical activity research conducted in other geographic regions, our findings are also consistent with previous non-GPS based studies of walking conducted in the Paris metropolitan area based on the large RECORD sample (>7000 participants) that showed that the spatial accessibility to services promotes recreational and transport walking [33,38]. 

A high spatial access to services may increase walking through a variety of pathways. First, increased access to destinations could increase the likelihood of walking to that destination. Notably, Walk Score primarily measures access to destinations. Second, it is possible that increases in neighborhood destinations may be related to pleasantness of the neighborhood, which may also be associated with increase walking. Our models were intentionally not adjusted for the distance covered in each trip, thus the documented Walk Score effect could reflect these two mechanisms (reduction of distance to cover in territories with high densities of services and pleasantness of the environment). If we were controlling for distance, we would block the mediating pathway through which people walk more in high Walk Score areas because they do not have to use a car to reach a service that they can find nearby their residence. 

It is also interesting to see that the associations with the trip origin and destination Walk Score documented with the survey walking outcome were also observed for the accelerometry-assessed number of steps, even if walking episodes identified with this accelerometer variable also refer to trips using other modes such as public transport.

Regarding other components of densities than services (e.g., buildings, street intersections, *etc*.), in previous studies related to the RECORD study, no associations were documented between the density of intersections and walking [30,38,45]. Walk Score is mostly a measure of spatial access to services, and future research should consider other components of densities, although this preliminary evidence that we have in the Paris region is that services are more influential than these other dimensions.

This study has implications for urban design and planning and for public health professionals. To increase physical activity and in particular walking, our results suggest that neighborhood design should aim to create walkable neighborhoods well-resourced in services. In addition to increasing the walkability of neighborhoods, behavioral interventions to promote neighborhood-based walking can use Walk Score (website and/or app) to show study participants amenities that promote walking such as parks in their neighborhood. Walk Score can also help neighbors to advocate for policies that would increase the walkability of their neighborhoods (e.g., zoning for mixed land uses, development of local parks) [25]. 

### 4.1. Future Research 

Future research with Walk Score and other measures of neighborhood walkability should be conducted to understand their potential connections to health behavioral outcomes such as walking. These future investigations should utilize a longitudinal research design to provide causal inference. We note though that some longitudinal research has reported associations between Walk Score and physical activity [46]. In addition, studies should be conducted across the European Union and other global geographic locations, including Asian and Africa. Beyond Walk Score, future studies can link Walk Score’s Transit Score (which has been validated) [41] and Walk Score’s new Bike Score (no validation on this bikeability metric has been conducted that we are aware of) to physical activity related outcomes, such as transit walking and biking. Emerging research suggests that neighborhood transit availability and bikeability can be associated with relevant physical activity outcomes [9,47,48]. Little work has been conducted using Transit Score and Bike Score in relation to health outcomes, but emerging research exists. For example, a recent study using data from the Multi-Ethnic Study of Atherosclerosis found that an increase in Transit Score was associated with lower odds of not transport walking or leisure walking, and additional minutes/week of leisure walking [9]. 

Further, it is possible that Walk Score (and other metrics of neighborhood walkability) could have different effects for different population groups. Assessing this was not the goal of the current study and the sample size was too small to differentiate effects between groups. Future research should examine how neighborhood walkability (including Walk Score) can influence walking outcomes by population subgroups. 

### 4.2. Study Limitations

This study is subject to limitations. First, this was a cross-sectional study. As such, while we found associations between Walk Score, transportation mode choice, and physical activity, these relationships may not be causal. An alternative explanation includes reverse causation and endogeneity. However, we note that emerging longitudinal research has reported relationships between Walk Score, walking, and body mass index [46]. Importantly, we note that in our previous work [30], we did not find endogeneity to be an issue in this particular GPS dataset. In this previous paper we adjusted for two neighborhood selection factors but it did not change at all the association [30], thus we decided to drop these variables from the present models. We also note that recent research suggests that the impact of neighborhood self-selection is minimal in studies of neighborhood built environments and physical activity outcomes [49]. Furthermore, we note that no Walk Score validation study has been conducted in international contexts, such as France (Walk Score has been validated in the U.S. only) [8]. However, we have no reason to believe that the Walk Score Paris data will be substantially different that in the U.S.-based studies and previous research has shown Walk Score is most valid in high population density areas [42]. This study used the available standard Walk Score data, which uses straight-line distances. The newer “Street Smart” Walk Score accounts for pedestrian friendliness factors (e.g., average block length) and therefore is likely more relevant to health outcomes; unfortunately it was not available to us for the Paris metropolitan area. However, previous research documented strong correlations between standard Walk Score and the Street Smart Walk Score [9]. Moreover, Transit Score and Bike Score in the Paris metropolitan area were unavailable to us at the time of data collection. In addition, Walk Score weights all destinations equally and does not consider certain neighborhood-related characteristics (e.g., crime). Several studies observed that built environment characteristics, including as measured by Walk Score, were positively associated with crime [39,50,51]. Consequently, residential confounding at the neighborhood level may be an issue, although we controlled for neighborhood education [52]. However, we note that residual confounding is an issue is all observational studies. We had a limited sample size for this GPS-based study. As such, this study might not be generalizable to all adult segments of the population of the Paris metropolitan area, or to adults in other cities and countries. However, many GPS studies have sample sizes of approximately 100 or fewer including recent GPS studies in urban areas such as New York City [53,54]. Future research will have to better account for the behavioral preferences of participants (preferences for certain places connected to preferences for certain modes) that may confound the trip-level associations between built environments and walking (previously referred as the selective daily mobility bias) [25,32]. Finally, we note that individuals in Paris often travel via the subway system and while they are underground GPS receivers are unable to obtain signals from GPS satellites, which may lead to data loss. It should be noted, however, that the impact of these losses should minimized by the GPS-based mobility survey that was conducted.

We determined trip-level contextual characteristics at the beginning and end of trips [55] but decided to not take into account environmental conditions along trip itineraries to avoid selective daily mobility biases [25], which can significantly compromise study results. Indeed, associations between exposures along trip itineraries and walking are likely confounded, because people select their exact itinerary according to their chosen mode (confounding by the preferences of participants). A potential way to address this concern would be to determine a number of itineraries between the origin and destination of each trip including the shortest one and other relatively short itineraries, and to aggregate exposure along these itineraries as an explanatory variable [25,30,55]. 

## 5. Conclusions

Walkable neighborhoods were associated with increases in walking among adults in Paris, as documented at the trip level. Creating walkable neighborhoods (including public health researchers and practitioners working with urban planners and community development organizations to change neighborhood design and increase commercial activity) may increase walking trips and therefore could be a relevant health promotion strategy, including to promote walking in neighborhoods and overall physical activity.

## Figures and Tables

**Table 1:** Number of trips per participant (made with all motorized and non-motorized modes) over 7 days according to categories of Walk Score.

Walk Score	Overall Number of Trips	Number of Trips Starting and/or Ending at the Residence
Average residential Walk Score	Mean (interdecile range)	Mean (interdecile range)
Very/Car-Dependent-Somewhat Walkable	27.0 (26)	17.8 (14)
Very Walkable	32.2 (33)	20.9 (20)
Walker’s Paradise	31.0 (29)	21.1 (20)
*p* for trend	<0.23 *	<0.07 *

* *p* values for trends in the number of trips across categories of residential Walk Score are based on the Jonckheere-Terpstra test.

**Table 2:** Distribution of transportation mode choice and walking according to the Walk Score (at the trip level).

Walk Score	Transportation Mode Choice (*n* = 6969) % (n)				
**Walk Score Trip Origin**	**Walking (Assessed in the Survey)**	**Bike**	**Public Transportation**	**Personal Motorized Vehicle**	**Number of Steps in the Trip per 10 min (*n* = 6313) Mean (±SD) (P25–P75)**
Very/Car-Dependent-Somewhat Walkable	16.0% (131)	2.8% (23)	6.1% (50)	67.6% (554)	221.4 ± 304.2 (30; 243.8)
Very Walkable	35.8% (724)	3.1% (61)	9.5% (193)	49.8% (1008)	350.7 ± 392.0 (41.0; 551.1)
Walker’s Paradise	52.9% (2182)	3.4% (138)	19.2% (790)	23.4% (967)	415.0 ± 378.7 (83.2; 691.5)
*p* for trend	<0.0001 *	<0.17 *	<0.0001 *	<0.0001 *	<0.0001 **
**Walk Score Trip Destination**	**Walking (Assessed in the Survey)**	**Bike**	**Public Transportation**	**Personal Motorized Vehicle**	**Number of Steps in the Trip per 10 min**
Very/Car-Dependent-Somewhat Walkable	16.0% (131)	3.5% (25)	6.6% (54)	67.4% (552)	235.4 ± 313.8 (35; 268.0)
Very Walkable	35.8% (725)	2.9% (59)	9.2% (187)	50.0% (1013)	347.9 ± 391.3 (39.7; 565.5)
Walker’s Paradise	52.9% (2181)	3.4% (138)	19.2% (792)	23.4% (964)	413.3 ± 378.7 (81.1; 691.8)
*p* for trend	<0.0001 *	<0.23 *	<0.0001 *	<0.0001 *	<0.0001 **

* *p* values for trends in the proportions of using a transportation mode by Walk Score category are estimated from the Cochran-Armitage test; ** *p* values for trends in the number of steps taken across Walk Score categories are estimated from the Jonckheere-Terpstra test.

**Table 3:** Trip-level associations between categorical Walk Score and walking in a trip, from multilevel logistic and linear adjusted for individual/neighborhood factors.

Walk Score	Walking in the Trip (Assessed from the Survey)		Number of Steps per 10 min	
	OR (95% CI); *N* = 6969		β (95% CI); *n* = 6313	
Walk Score-Trip Origin				
(*vs*. Very/Car-Dependent/Somewhat Walkable)				
Very Walkable	2.36	1.84 to 3.02	+79	+46 to +112
Walker’s Paradise	3.48	2.72 to 4.44	+91	+58 to +124
Walk Score-Trip Destination				
(*vs*. Very/Car-Dependent/Somewhat Walkable)				
Very Walkable	2.36	1.85 to 3.03	+56	+23 to +89
Walker’s Paradise	3.48	2.73 to 4.44	+68	+35 to +101

Notes: The level 2 (individual-level) variance was of 0.96 (95% CI: 0.93, 0.99) in the logistic model for walking in the trip and of 118128 (95% CI: 114,040, 122,442) in the linear model for the number of steps taken per 10 min.
